# Sharpening our understanding of saber‐tooth biomechanics

**DOI:** 10.1002/ar.25690

**Published:** 2025-05-22

**Authors:** Tahlia Pollock, Philip S. L. Anderson

**Affiliations:** ^1^ Palaeobiology Research Group The University of Bristol Bristol UK; ^2^ School of Biological Sciences Monash University Melbourne Victoria Australia; ^3^ Department of Evolution, Ecology, and Behaviour School of Integrative Biology, University of Illinois Urbana‐Champaign Urbana Illinois USA

**Keywords:** biomechanics, functional morphology, puncture mechanics, tooth mechanics, tooth morphology

## Abstract

Saber‐teeth are a striking example of convergent evolution in vertebrate predators, having evolved multiple times in mammals and their early ancestors. While there is broad consensus that saber‐toothed taxa employed a distinct biting strategy compared to conical‐toothed carnivores, like the lion, the precise mechanics and variability of this bite remain debated. In this review, we integrate current knowledge of pointed tooth mechanics and puncture mechanics to explore predatory function, focusing on the canine shear‐bite hypothesis. We quantify the key morphological characteristics of saber‐teeth–elongation, slenderness, curvature, sharpness, and cross‐sectional shape in a sample of saber‐and conical‐toothed taxa. Using the morphological diversity observed and insights from experimental studies, we examine the capacity of saber‐teeth to perform the canine shear‐bite, contrasting them with the clamp‐and‐hold bite of extant carnivores with conical canines. Our findings indicate that the morphological characteristics associated with extreme saber‐tooth forms, as seen in *Smilodon*, suggest the prioritization of deeper puncture and slicing actions and limiting of lateral loads, favorable for a canine shear‐bite. However, we also demonstrate that these morphological characteristics exist on a continuum accross saber‐toothed taxa suggesting greater functional diversity beyond the shear‐bite versus clamp‐and‐hold bite dichotomy. While this study refines our understanding of saber‐tooth function, key gaps remain, particularly regarding the role of cross‐sectional shape, curvature, and serrations in puncture mechanics.

## 1 INTRODUCTION

### 1.1 Saber‐tooth problem

The iconic “saber teeth” first appeared ~265 million years ago in the Permian and have been present in ecosystems around the globe until as recently as the last ice age ~10,000 years ago (Bocherens et al., 2016; Van Valkenburgh & Jenkins, 2002). During this time these elongated canine teeth have evolved at least five times in mammals and their early relatives and are found in a variety of carnivorous taxa including the therapsid gorgonopsians, eutherian “cat‐like” carnivorans like the creodonts, nimravids, and barbourofelids, machairodont felids like the infamous *Smilodon*, and even *Thylacosmilus* a metatherian sparassodont; none of which survive to the present day (Lautenschlager et al., 2020; Martin et al., 2000; Van Valkenburgh & Jenkins, 2002). With no living analogues, determining how exactly these extreme canines may have been used presents a challenge. One that has been subject to much debate over the years, having been discussed, re‐assessed and re‐interpreted many times (Akersten, 1985; Andersson et al., 2011; Antón et al., 2004; Antón & Galobart, 1999; Biknevicius et al., 1996; Chatar et al., 2021, 2022, 2024; Christiansen, 2007; Christiansen, 2008; Christiansen, 2011; DeSantis et al., 2012; Deutsch et al., 2025; Duckler, 1997; Emerson & Radinsky, 1980; Figueirido et al., 2018; Figueirido et al., 2025; Janis et al., 2020; Lautenschlager et al., 2020; McHenry et al., 2007; Melchionna et al., 2021; Piras et al., 2018; Pollock et al., 2025; Shelbourne & Lautenschlager, 2025; Simpson, 1941; Slater & Van Valkenburgh, 2008; Therrien, 2005; Van Valkenburgh, 2007; Van Valkenburgh, 2008; Van Valkenburgh & Hertel, 1993; Van Valkenburgh & Ruff, 1987; Werdelin et al., 2018; Wroe, 2008; Wroe et al., 2013). Numerous hypotheses have been proposed for exactly how the saber teeth of these predators were used to kill and dismember prey that relate to the mechanics of the killing bite (Akersten, 1985; Andersson et al., 2011; Antón & Galobart, 1999), the relative contribution of jaw adductor versus head depressor muscles (McHenry et al., 2007; Wroe et al., 2013), the bite forces they can generate and withstand (Christiansen, 2007, 2011; Therrien, 2005; Van Valkenburgh & Ruff, 1987), the area on the prey targeted (Duckler, 1997), the size of the prey targeted (Andersson et al., 2011), and the strength of their forelimbs and the degree to which they were used to immobilize prey before biting (Meachen‐Samuels & van Valkenburgh, 2010). Broadly, there is a general consensus that saber‐toothed taxa targeted relatively large prey and delivered slashing bite(s) (something like the canine shear‐bite (Akersten, 1985)) to the soft tissue of the throat that are predominantly driven by head depressor muscles. This appears to exist on a continuum between specialized saber‐toothed predators like *Smilodon*, which have been attributed the canine shear‐bite and extant conical tooth cats like the lion (*Panthera leo*), which employ a clamp‐and‐hold bite (Chatar et al., 2022; Chatar et al., 2024; Ewer, 1973; Figueirido et al., 2018; Figueirido et al., 2025; Lautenschlager et al., 2020; Melchionna et al., 2021; Wroe et al., 2013) (Pollock at al. 2025). Despite this, there is still controversy, like in the recent study from (Janis et al., 2020) that proposes *Thylacosmilus* likely did not use its saber teeth to dispatch prey, as is suggested for *Smilodon*, but rather used them for opening and disemboweling carcasses.

Almost all previous assessments of sabretooth killing behavior are based on morphological or biomechanical analyses undertaken on the skull and teeth as an integrated unit. There are exceptions to this, like Van Valkenburgh and Ruff (1987) modeling relative strength in mammalian carnivore canines via beam theory, a follow‐up study conducted by Christiansen (2007) which also incorporated dry skull estimates of bite force, and a more recent study testing whether saber‐teeth represent functional optimal designs (Pollock et al., 2025). Others we present here in this *Special Issue* include a morphofunctional study from Shelbourne and Lautenschlager (2025) and a theoretical ontogenetic study from Tseng (2025). Such studies provide valuable insights into the tooth in question: the saber.

In this review, we synthesize our current understanding of the functional biomechanics of pointed teeth to examine predatory ecology. Asking the question: “what can pointed tooth biomechanics tell us about saber‐teeth?” This will involve discussing tooth mechanics and puncture mechanics, then identifying variables (aspects of tooth form) that are relevant to saber‐teeth. We will examine the morphological diversity of saber‐teeth across a sample of taxa and compare them to key examples of their conical toothed counterparts. Then we will integrate the observed morphological diversity with our understanding of puncture and tooth mechanics to examine key hypotheses of how these predators may have used their iconic teeth, such as the canine shear‐bite hypothesis.

### 1.2 Tooth mechanics

Mammalian teeth have been a subject of study for decades in both biology and palaeobiology, with a great deal of work dedicated to understanding how tooth form and function relate to potential ecology, see Crofts et al. (2020); Evans and Pineda‐Munoz (2018) for comprehensive reviews. The ecology of mammalian dentition is of particular interest in palaeobiology for several reasons: (1) Teeth are often the only part of the animal that fossilizes. There are many more mammalian teeth in the fossil record than there are full skeletons. (2) We cannot observe fossil organisms eating, so we need some way to (indirectly) assess their ecology. While gut contents can sometimes be found, it is always worth remembering that gut contents simply tell you about the animal's last meal, not necessarily what they ate normally. (3) Teeth sit right at the interface of the animal and its environment (Ungar, 2010). They help animals to access the nutrients required to function and survive and can be viewed at tools with the principal function of food breakdown (Evans et al., 2007; Evans & Pineda‐Munoz, 2018; Evans & Sanson, 2003). As such, they offer valuable information about an animal's ecology which we can leverage to better understand saber‐tooth forms.

Several researchers have attempted to discern diet from the wear patterns left on teeth at both the macro and micro structural levels (DeSantis, 2016; Van Valkenburgh & Hertel, 1993). An example can be found in this Special Issue, where Pardo‐Judd and DeSantis (2025) use dental microwear texture analysis to demonstrait consistent feeding behaviour in *Smilodon*. While these methods have proven incredibly useful for identifying potential ecological categories across mammals and even identifying ecological niches in fossil forms, they do not necessarily aid in understanding the evolution behind the tooth forms themselves or how exactly they were used. To better understand the potential evolutionary and adaptive drivers of tooth form, many researchers have attempted to quantify tooth forms in ways that allow for easy comparisons between taxa. Early studies relied on analogous structures, assuming similar forms processed similar foods, with features like molar crests measured and dietary inferences drawn from the degree of similarity or difference observed (Strait, 1993). These comparative studies rely on similarity in shape of the tooth structure to an extant species with known ecology to compare tooth features in a meaningful way. This approach is limited for extinct species, as teeth are often the only preserved remains, and modern analogues are lacking for forms like saber‐teeth.

Other studies have attempted to create a more objective way of quantifying tooth form that is informed by mechanical theory and relates it directly to ecology (Evans et al., 2007; Lucas, 1982; Lucas & Luke, 1984; Popowics & Fortelius, 1997; Van Valkenburgh & Ruff, 1987). Aspect(s) of a tooth form with known functional implications can be quantified and then compared across a range of taxa to infer ecology/likely foods processed. The approach is based on our understanding of mechanical theory and the principles that govern the deformation and fracture of food materials, which vary significantly across animals (e.g., plant tissues like grasses, leaves, fruits, and nuts to animal tissues like muscle, hide, bone, exoskeleton, and even shell). Food materials differ in their fundamental microstructure, their material response to loads, and their fracture mechanics (Anderson, 2018; Crofts et al., 2020; Evans & Pineda‐Munoz, 2018; Lucas, 2004). As material properties vary, so do the tooth forms that are most effective at breaking them down.

As an example, we can investigate the properties of meat, bone/exoskeleton, and plant tissues to see how these properties dictate the most effective tooth shapes for breaking them down. Muscle and hide are relatively ductile materials as they will transmit any applied force into deformation and, as a result, will readily deform when force is applied (Anderson, 2018; Crofts et al., 2020; Freeman & Lemen, 2006). They are also tough, meaning that once a crack is initiated, a significant amount of energy needs to be imparted to propagate it through the material (Anderson, 2018; Crofts et al., 2020; Evans & Pineda‐Munoz, 2018; Lucas & Luke, 1984). These materials require sharp tips that concentrate the force over a smaller surface area, which creates high localized stress to initiate cracks in the prey, as well as sharp blades to continuously apply force to propagate cracks through the prey (Anderson, 2018; Crofts et al., 2019, 2020; Evans & Sanson, 1998; Evans & Sanson, 2003; Freeman & Lemen, 2006; Lucas, 1982; Shergold & Fleck, 2005). Bone and exoskeleton are relatively stiff materials as they store the energy applied to them; as a result, they have a very small degree of deformation when force is applied. They are also somewhat brittle, and once initiated, cracks more readily propagate and require less input of energy (Anderson, 2018; Crofts et al., 2020; Lucas, 2004). Blunt tooth surfaces and robust teeth are better able to cope with the high forces and unpredictable loads encountered when biting into these materials (Lucas & Luke, 1984; Popowics & Fortelius, 1997; Van Valkenburgh & Ruff, 1987). This is because, when biting, the force is being spread over a larger cross‐sectional area of tooth, which produces lower stresses in the tooth, likely reducing the risk of breakage (Evans & Sanson, 1998; Freeman & Weins, 1997). Plant materials, leaves, grasses, and stems can be considered fibrous and tough; here, greater occlusal complexity (multi‐cusped teeth with many blades) facilitates breakdown by increasing the points of contact to initiate and propagate cracks (Evans et al., 2007).

Based on these relationships between material properties and tooth morphology, it is reasonable to infer that saber‐teeth were adapted for targeting ductile and tough materials, such as muscle and flesh. However, something is still missing from these studies based on mechanical theory, which is a direct link between aspects of tooth form and the actual biomechanics at play during feeding.

This is where biomechanical studies are integral; they allow researchers to quantify the functional capabilities and performance of biological systems and structures, which can involve the creation of digital or physical models to determine if and how structures are able to carry out specific functions. Two particular biomechanical approaches, finite element analysis (FEA) and physical performance testing, have been extensively applied to teeth (Abler, 1992; Anderson & LaBarbera, 2008; Berthaume et al., 2010; Crofts & Summers, 2014; Dumont et al., 2005; Evans & Sanson, 1998; Freeman & Lemen, 2006, 2007a, 2007b; Torices et al., 2018; Whitenack & Motta, 2010) (Ballell at al., 2022; Pollock et al., 2022).

FEA was originally developed to predict the behavior of manufactured structures or objects like bridges and car parts and utilizes engineering principles to create and test digital models (either 2D, 2.5D, or 3D) of structures in simulated functional scenarios (Zienkiewicz & Taylor, 2000). Briefly, a model of the structure in question is simplified into a volume mesh containing a number of “finite elements,” for example, tetrahedrons, that are connected to one another via their vertices or “nodes” (Dumont et al., 2009). This mesh is then assigned specific material properties, which are selected based on the material of the structure simulated. The model is then constrained (areas of the structure that need to be fixed in position) and loaded (magnitude, distribution, and direction of forces to be applied to the structure). Given these input variables, the computer runs the simulation and solves the model, mapping how stresses and strains are distributed within the structure (Rayfield, 2007; Richmond et al., 2005). The resulting stress and strain output helps to quantify how the structure responds to the specific simulated functional scenarios. If we consider pointed teeth specifically, FEA has been used to investigate the function of serrations in theropod teeth (Torices et al., 2018), model puncture in snake fangs (Rajabizadeh et al., 2021), quantify how aspects of shape impact feeding biomechanics in carnivoran canines (Freeman & Lemen, 2007a; Pollock et al., 2022; Shelbourne & Lautenschlager, 2025), compare the functional capabilities of extant and extinct shark teeth (Ballell & Ferrón, 2021), infer diet in early dinosaurs (Ballell et al. 2022), examine how tooth biomechanics changes throughout eruption (Tseng, 2025), and test hypotheses related to the presence of ridges in marine tetrapods (MacKenzie et al., 2024) and mammals (Anderson et al., 2011). Such studies have provided new insights into relationships between tooth strength/resistance to breakage and test aspects of the mechanical theory that underlies them. Despite their utility, simulation‐based approaches must be validated, and this is where physical performance testing plays a critical role.

Physical performance testing measures the behavior of a biological structure via physical experiments. Most often, a force tester is used to measure performance variables like maximum force (Newtons) and energy (Newtons × Displacement (mm)) required to puncture, crush, cut, pull, tear, or break a substrate or structure. In the context of teeth, it enables researchers to assess the influence that both the functional parameters of a tooth and the structural properties of a food have on the performance of a particular tooth form. Performance testing is particularly useful in the assessment of teeth, with mechanisms easily set up to mimic natural forms of biting or occlusion, for example, a simple punch (single tooth), guillotine (single blade) or scissors (double blade) (Anderson & LaBarbera, 2008). Theoretical models of teeth have generally been used, for example, simplified blades or cusps to represent tooth forms of extant and extinct carnivores, testing the functional performance of serrations, notches, and other aspects of shape (Abler, 1992; Anderson, 2009; Anderson & LaBarbera, 2008; Crofts et al., 2019; Crofts & Summers, 2014; Evans & Sanson, 1998; Freeman & Lemen, 2006, 2007b; Freeman & Weins, 1997). By undertaking a series of physical tests with punches that varied in sharpness, Evans and Sanson (1998) were able to demonstrate experimentally that sharper tips reduced the force required to puncture beetle prey. In another study, Anderson and LaBarbera (2008) employed cutting tests with simplified blades to show experimentally that notched dentitions reduced the work to fracture tough materials. Studies that employ theoretical models are useful as they are able to tightly control parameters to quantify the impact of key aspects of tooth shape. This is because they help untangle the covariation of aspects of shape, which can limit the investigation of natural forms (Pollock et al., 2022). While useful for isolating variables, these models are not accurate representations of real tooth morphology. Hence, recent studies have used direct copies of teeth to quantify performance in a variety of contexts: puncture, draw feeding in sharks, crushing abilities of hominin molars, and even material response in starch‐based pet foods (Berthaume et al., 2010; Skamniotis, 2017; Skamniotis et al., 2017; Whitenack & Motta, 2010). Some studies even use actual teeth (Corn et al., 2016; Crofts et al., 2019; Crofts & Anderson, 2023; Freeman & Lemen, 2007b; Goldschmidt et al., 2017; Soukup et al., 2015). More recently, real and theoretical tooth forms have been used in combination to build a more comprehensive picture (Pollock et al., 2024). A large proportion of these physical tests focus on cutting or crushing. Studies of the mechanics of puncturing teeth in mammals have been even more scarce than cutting mechanics. Some work has been done showing how bat canines balance being long and thin enough for successful puncture while still being robust enough to prevent breakage (Freeman & Lemen, 2007b). Other experimental work has focused on specific aspects of tooth shape (Evans & Sanson, 1998) or wear (Pollock et al., 2024) to understand how variables influence performance. Beyond just mammals, work has been done on the puncture mechanics of other vertebrate teeth (Crofts et al., 2019; Crofts & Anderson, 2023; Whitenack & Motta, 2010) as well as in invertebrates (Schofield et al., 2016).

These experimental studies, when coupled with the FEA modeling mentioned above, give us a potential roadmap for trying to understand the mechanics of puncture teeth in other animals, such as fossil sabertooth mammals. Understanding the mechanics of saber‐teeth as specialized puncture tools provides a unique opportunity to apply these principles in interpreting saber‐tooth function.

### 1.3 Puncture mechanics

Before we can apply puncture mechanics to the question of saber‐tooth function, we need to define puncture and outline what we understand about the mechanisms that govern successful puncture. Puncture occurs when a tool is used to create a fracture in a target material, followed by penetration of the tool into the material (Anderson, 2018; Zhang & Anderson, 2022). This definition of puncture is in sharp (pun intended) contrast with the crushing/cutting mechanics associated with mastication that have been the focus of previous work on vertebrate dentitions. The goal of mastication is to reduce food items from one large piece to multiple smaller pieces for easier digestion; this can occur via either crushing brittle foods (nuts, shells, etc.) into small pieces or cutting/tearing softer materials (muscle or skin). Puncture, as defined above, leaves the target coherent, allowing the tool to be inserted within it.

While leaving the target coherent may seem counter‐productive, there are actually several functions that puncture allows in biological organisms (see Anderson (2018) for a full review). Puncture can be used to capture prey items prior to ingestion, either via the use of two opposing tools, such as the canine teeth of carnivorous mammals or using one tool as a harpoon, as seen in mantis shrimp. Puncture is also used to inject substances into targets, such as venom in snakes or even eggs, as in parasitoid wasps. Some puncture tools even aid in reproduction, such as the clinging spines of colonially reproducing cacti or the hooked spines found on chondrichthyan pelvic claspers. Regardless of the biological functions, all these examples must overcome similar challenges to perform puncture.

While there have been several studies over the years testing puncture performance in specific biological systems, it is only recently that researchers have attempted to examine how size, shape, and materials influence puncture performance across taxa. Experimental work over the last decade has illustrated the relationship between puncture performance and several variables such as morphological sharpness (Crofts et al., 2019; Freeman & Lemen, 2006; Pollock et al., 2024), tool curvature (Zhang et al., 2024), surface ornamentation (Cho et al., 2012; Crofts & Anderson, 2018), material deformation and density (Anderson et al., 2019) and dynamic energy (Anderson et al., 2016; Zhang & Anderson, 2024). A recent paper even shows how these variables interact, revealing that the influence of tool morphology on puncture efficiency lessens at higher impact speeds (Zhang & Anderson, 2023). Data from these experimental papers has also been used to build models which attempt to contextualize a framework for puncture mechanics in terms of scale and energy (Schofield et al., 2016; Zhang & Anderson, 2022). Specifically, a recently published mathematical model highlighted several variables that have a strong influence on the energetics of puncture (Zhang & Anderson, 2022). While more work is still required to fully understand the material response to dynamic impacts, these models offer a great framework for examining the form–function relationship of piercing teeth such as those found in saber‐tooth forms.

### 1.4 Identifying variables

If we want to explore the potential puncture mechanics of extinct forms such as saber‐toothed mammals, we need to identify a set of variables/parameters that can be assessed from fossilized remains and have a strong connection to puncture performance. Here we identify a series of potential variables and define their relation to puncture. Puncture will be discussed in terms of the act of the tool penetrating a material and the ability of the tool to resist breaking during puncture and how shape parameters impact both of these variables.

### 1.5 Cusp angle/taper

The cusp angle or taper of a puncture tool determines whether the tool is long and thin, or shorter and fatter. Measurements relating to this feature have been explored in several groups including extant mammalian canines (Evans et al., 2021; Evans & Sanson, 1998; Freeman & Lemen, 2007b; Pollock et al., 2022) as well as snake fangs (Cleuren et al., 2021; Crofts et al., 2019; Palci et al., 2023; Segall et al., 2023) and in invertebrate forms (Schofield et al., 2016). The approaches to measuring this trait differ nearly as much as the diversity of taxa in which it appears. Some involve looking at the volume or surface area of the puncture tool a standard length from the tip. Others attempt to be scale independent, using the ratio of base to height or determining the angle made by the two sides of the cone. More recently, the Power Cascade model (Evans et al., 2021), which mathematically describes the shape of pointed structures using a power law function.

How ever, this trait is measured, it has been shown to be a key variable when it comes to understanding puncture mechanics. Previous studies have identified correlations between tool slenderness and the puncturing of ductile and tough materials, for example, in carnivore canines (Pollock et al., 2022), snake fangs (Cleuren et al., 2021; Crofts et al., 2019), and marine tetrapods (Fischer et al., 2022). Such patterns have been verified experimentally with physical tests, where slender tooth models were found to require less force to puncture than robust models (Crofts et al., 2019; Evans & Sanson, 1998; Pollock et al., 2024; Zhang & Anderson, 2023). Current models of puncture performance, backed up by experimental and theoretical studies, have shown taper to be a key determiner of puncture depth in both static and dynamic situations (Zhang & Anderson, 2022, 2023). Having a long slender tool allows for more efficient puncture due to a reduction in the amount of energy spent pushing apart tissues and a smaller surface area for accumulating frictional energy. At the same time, taper has been shown to be a key factor in resistance to bending/breakage in biological structures (Pollock et al., 2022; Quan et al., 2024), whereby, for a given load, slender forms experience higher stresses and are more likely to break than robust forms. Such patterns illustrate a trade‐off between puncture performance and the ability to withstand breakage (Freeman & Lemen, 2007b; Pollock et al., 2025).

### 1.6 Tip sharpness

Tip sharpness is primarily involved in the initiation of puncture. By having a small contact area between the tip of the tool and the target surface, more stress can be accumulated for a given force, allowing for higher stresses. Once the critical stress (determined by a combination of material properties and scaling) is reached, fractures are propagated. Similar to cusp shape, there are several ways of determining tip sharpness. In the literature, it has been measured in a myriad of ways including surface area or volume, cross‐sectional area, taper and aspect ratio, tip diameter, tip included angle, and radius of curvature (Cleuren et al., 2021; Crofts et al., 2019; Evans & Sanson, 1998; Frazzetta, 1988; Freeman, 1992; Freeman & Lemen, 2006; Hartstone‐Rose & Wahl, 2008; Jones et al., 2012; Lucas, 1982; Pollock et al., 2024; Popowics & Fortelius, 1997). Among these varied metrics, there are broadly two types of sharpness that can be quantified: absolute sharpness and relative sharpness. Absolute sharpness refers to the original unscaled value of sharpness measured, whereas relative sharpness refers to the value of sharpness that has been scaled by a particular factor, for example, body size (Popowics & Fortelius, 1997); Evans et al. (2005), tooth height (Cleuren et al., 2021), cross‐sectional area (Pollock et al., 2022) or is scale independent, for example, to take an angle of the tip (included angle), which has been shown in snake fangs to be a slightly better predictor of performance (Crofts et al., 2019).

Tip sharpness, while a morphological feature, can be difficult to assess in fossil forms. In particular, the effects of wear on tip sharpness have been shown to have a large influence on puncture mechanics (Pollock et al., 2024), especially in mammals where there is no tooth replacement to keep the tips pristine. Furthermore, in fossil specimens, we can never be sure whether the tips we find are a true reflection of the original morphology, or if aspects of the fossilization process have altered them. As such, tip sharpness needs to be assessed with caution across fossil groups.

### 1.7 Cross‐sectional shape and degree of lateral compression

An often‐overlooked aspect of puncture tool design is the shape of the cross section. Very few biological tools have completely conical puncture tools; more often than not, they are flattened to some extent and exhibit more ovoid laterally compressed cross sections. How this flattening of the cross section influences puncture is not entirely clear, but certain hypotheses can be made. A flatter tool can slide into a narrower fracture opening, allowing for insertion with a reduction in the amount of deformation of the target material required. On the other hand, flatter tools will have greater surface area relative to conical ones of the same volume, leading to greater frictional resistance (Zhang & Anderson, 2022). How these competing aspects of shape relate to puncture is still to be tested, but the cross‐sectional shape of the tool will likely have a large influence on puncture performance. From previous work modeling lateral bending in pointed teeth, we know that tool cross‐sectional shape impacts strength in particular directions (Christiansen & Wroe, 2007; Pollock et al., 2022; Tseng, 2025; Van Valkenburgh & Ruff, 1987). Rounder cross sections are better able to tolerate multidirectional loads, while more flattened/laterally compressed ovoid cross sections are better able to tolerate high stresses along their longest axis.

Cross‐sectional shape can vary beyond simple flattening. Puncture tools often have more unusual cross sections, such as the T‐shaped cross sections seen in stingray spines. In pointed teeth specifically, we observe a range of cross‐sectional shapes that can vary from simple circles and ellipses to outlines that are subrectangular, triangular, lenticular, or resemble a figure of eight (Freeman, 1992; Freeman & Lemen, 2006; Hendrickx et al., 2015). See fig. 5 in Hendrickx et al. (2015) for a comprehensive range of those observed in theropod dinosaurs. These cross‐sectional shapes will vary with the presence, absence, number of, or positioning of edges along the length of the tooth, which brings us to another important variable to consider: edges and their sharpness (Freeman, 1992; Pollock et al., 2022).

### 1.8 Edge(s) and surface structures

Many pointed teeth have blade‐like edge(s) along the tool including bat canines, carnivoran canines, snake fangs, and dinosaur teeth (Cleuren et al., 2021; Freeman, 1992; Hendrickx et al., 2015; Pollock et al., 2022). In mammals, the presence and sharpness of such edges are associated with diets that have a higher proportion of flesh and meat (Pollock et al., 2022) Having one or more blade‐like edges along the tool could have the effect of allowing for a certain amount of slicing action during puncture, which depending on the material punctured, will impact performance (Freeman, 1992). In a study using simplified edged and non‐edged punches, Freeman and Lemen (2006) demonstrated that the presence of sharp edges decreased penetration force in tough materials like leather and turkey skin, and ductile materials like chicken flesh. Here, the sharp edges act to create new cracks (relative to a smooth punch) and provide the continued contact necessary to help propagate them in the tough and ductile materials (Crofts et al., 2020; Freeman, 1992; Freeman & Lemen, 2006). While blade‐like edges can enhance puncture efficiency and slicing capabilities, the addition of surface structures like serrations and imbrications also impacts puncture mechanics, influencing both penetration and extraction.

Many puncture tools have serrations, imbrications, ridges, or other such textures that can have multiple effects. In both porcupine quills and cactus spines, it has been shown that similar imbricated textures can both improve puncture efficiency and make it much harder to extract the tool after insertion (Cho et al., 2012; Crofts & Anderson, 2018). In teeth specifically, surface structures like the apico‐basal ridges observed in marine tetrapods have been hypothesized to aid in puncture and extraction (McCurry et al., 2019), and in a recent study were shown to have no effect on tooth strength (MacKenzie et al., 2024). Serrations, such as those found on archosaur teeth, have been hypothesized to aid in tearing food (Abler, 1992), but will also influence puncture in as yet uncertain ways.

When aligned along the edges of pointed teeth during puncture, serrations can help aid the slicing that occurs during puncture. This was demonstrated experimentally by Abler (1992), who showed that adding serrations along bladed edges improves a tool's ability to “grip‐and‐rip” during cutting, reducing reliance on concentrated applied force. This has also been hypothesized for shark teeth, whereby serrated teeth can maximize the available biting forces and enhance their cutting ability compared to smooth‐edged teeth (Frazzetta, 1988). Serration morphology can differ too (e.g., sharks: (Moyer & Bemis, 2017), theropod dinosaurs: (Hendrickx et al., 2015), hypercarnivorous synapsids and dinosaurs: (Whitney et al., 2020)), which likely also has implications for function; however, no study has experimentally tested this. To formulate hypotheses of how serration morphology might impact performance, we can look to man‐made tools like saw blades. For example, blades used for meat cutting can be scalloped (designed for boneless cuts of meat) or saw‐toothed (designed for cuts including bone) (Moyer & Bemis, 2017). Moyer and Bemis (2017) provide a comprehensive look at serrations in three species of shark. See especially Figure 7 in Moyer and Bemis (2017) illustrating key saw‐blade characteristics from the manufacturing industry. Regardless of their form, surface elements must be considered along with the overall morphology of the puncture tools.

### 1.9 Curvature

Similar to cross‐sectional shape, the curvature of a puncture tool has not been carefully examined in terms of puncture performance but is a key morphological variable across saber‐toothed forms. The limited studies looking at tooth curvature and function have identified links between curvature and prey capture and retention in a range of predatory animals like snakes, sharks, and some seals (Frazzetta, 1966, 1988; Kardong, 1980; King, 1983; Reidman, 1990). Except for (Kajiura & Tricas, 1996), no study has tested the biomechanical implications of increased curvature on retention. Unlike the other traits mentioned, it is less intuitive how curvature might influence puncture performance. Only one study has investigated the impact of tool curvature on puncture performance, with surprising results. Via an integrated approach, combining puncture experiments, FE damage simulation, and energetic theory, Zhang et al., (2024) were able to demonstrate experimentally that structural curvature plays a minimal role in the efficiency of biological puncture. Having a highly curved tooth could be an indication that the vector of motion during puncture is not straight. You would not want to try to push a raptor claw straight into a substrate; it would make more sense for the motion to be rotational, allowing the puncture to occur in an arc that matches the shape of the claw itself. Note that the majority of biological puncture systems are rotational in nature, particularly teeth in jaws which rotate to close. The curved nature of many fangs/canines may simply reflect the motion of the teeth as the jaws close. Taken this way, curvature does not aid puncture so much as it ensures that the puncture tool is always aligned with the vector of motion, which previous work has shown to be optimal for decreasing tooth stress during biting (Pollock et al., 2022).

## 2 METHODS

### 2.1 Sample

**FIGURE 1:**
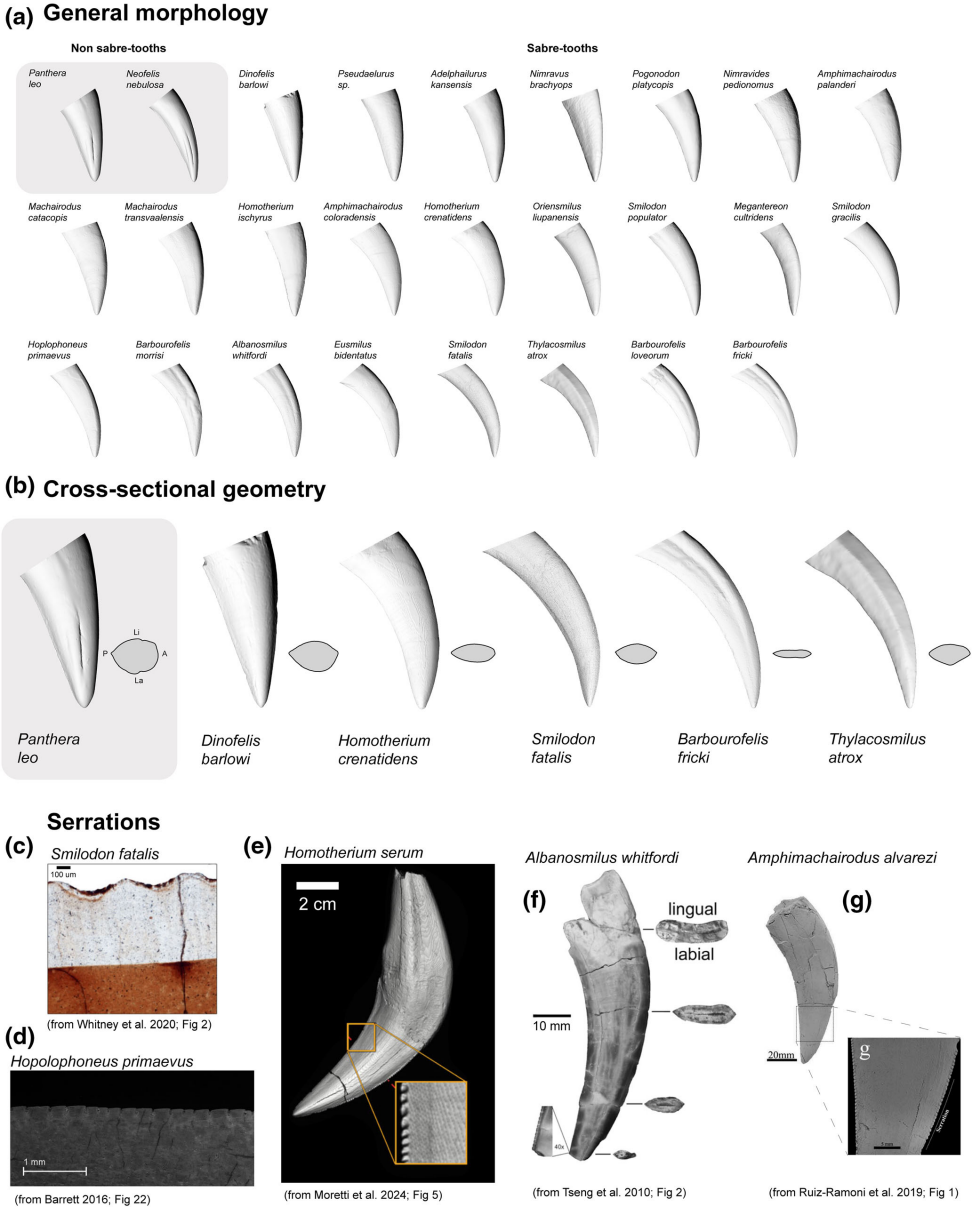
Diagram illustrating key aspects of saber‐tooth morphological diversity. (a) Labial views of all saber and non saber/conical toothed taxa measured in this study. (b) Labial and cross‐sectional views of a selection of teeth measured in this study; A = anterior, P = posterior, Li = lingual, and La = labial. (c)–(g) images from previous studies illustrating serrations in a selection of saber‐tooth forms. (c) *Smilodon fatalis* from (Whitney et al., 2020), (d) *Hoplophoneus primaevus* from (Barrett, 2016), (e) *Homotherium serum* from (Moretti et al., 2025), (f) *Albanosmilus whitfordi* from (Tseng et al., 2010), and (g) *Amphimachairodus alvarezi* from (Ruiz‐Ramoni et al., 2020).

We now have a detailed framework for understanding the mechanics of pointed puncturing teeth. Equipped with this toolkit, we can examine how these principles apply to the morphology of saber teeth. To begin, we will explore the morphological diversity observed in a representative sample of saber‐toothed taxa. Here, we feature a sample of sabretooth (24) and non saber‐tooth/conical tooth (2) forms to illustrate the morphological variation present in the key anatomical features outlined above. Our sample consists of 24 species including members of the Felidae (14), Nimravidae (9), and Thylacosmilidae (1) families (Figure 1; supplementary Data S1). Our dataset is also supplemented with images from previous works (Barrett, 2016; Moretti et al., 2025; Ruiz‐Ramoni et al., 2020; Tseng et al., 2010; Whitney et al., 2020) to examine serrations.

### 2.2 Quantifying tooth morphology

Some aspects of tooth morphology were compared qualitatively, including cross‐sectional shape and the presence or absence of surface structures like serrations. Others were compared quantitatively, such as cusp angle/taper, tip sharpness, curvature, and the degree of lateral compression/flattening. These aspects of shape were measured in Rhinoceros 5 (McNeel North America, Seattle, WA, USA), via both pre‐existing and new methods.

**FIGURE 2:**
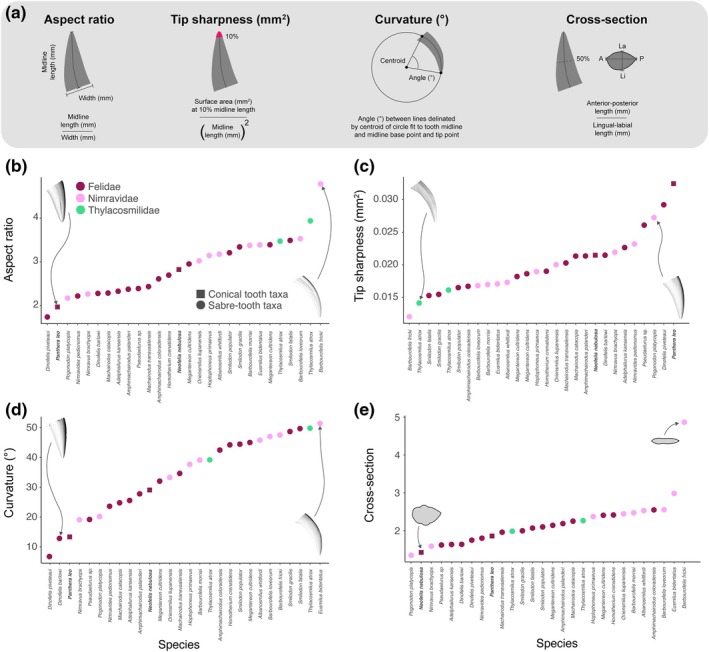
Diagram of key aspects of saber‐tooth morphology and how they vary among species. (a) Diagrams showing how each tooth shape metric (aspect ratio, tip sharpness, curvature, and cross‐section ratio) was measured. Aspect ratio was calculated as the length of the tooth midline divided by the width of the tooth at its base. Tip sharpness was calculated at the scaled surface area of the tip of the tooth at 10% of the midline length. Curvature was calculated as the angle between two lines, one defined by the centroid of a circle fit to the midline of the tooth and the base point of the midline and the other between the centroid and the tip of the tooth. Cross‐section ratio was calculated as the anterior–posterior length of the tooth divided by the lingual‐labial length of the tooth. (b) shows the variation in tooth aspect ratio among non saber‐tooth and saber‐tooth taxa, (c) the variation in tip sharpness, (d) the variation in tooth curvature, and (e) the variation in cross‐section ratio.

Cusp angle/taper was measured as the ratio of tooth length to width, here termed aspect ratio. The length of each tooth was delineated by the midline, a line that was defined by making a cross‐section through each tooth along the anterior–posterior axis and then calculating the midline between the anterior and posterior section lines. The length of the midline was then divided by the width of the tooth at its base, which was defined at the point where it intersects with the jaw (see Figure 2 for details). Higher values describe teeth that are longer and thinner, and lower values describe teeth that are shorter and thicker.

Tip sharpness was measured as the scaled surface area of the tooth tip at 10% of the midline length. This was calculated by measuring 10% of the length of the midline (starting at the tip of the tooth and moving toward the base) and using it to make a perpendicular cut through the tooth. We then calculated the surface area of the tooth tip; this method is based on the protocol from (Pollock et al., 2024). To enable comparison among species with teeth of very different tooth sizes, we scaled the surface area of the tooth tip so that: surface area (mm^2^) at 10% midline length/(Midline length (mm))^2^(see Figure 2 for details). Higher values describe teeth that have blunter tips, and lower values describe teeth that have sharper tips.

Curvature was measured as the angle between two lines; these lines were delineated by the centroid of a circle fit to the tooth midline and midline base point and tip point. This was calculated by fitting a circle to the midline of each tooth. This circle was generated from three points, one at the base of the midline, one at the tooth tip, and one in the middle of these two points. The centroid of this circle was then calculated and used to define two lines, one drawn between the centroid and the base of the midline, and the other between the centroid and the tooth tip. Tooth curvature was defined as the angle between these two lines (see Figure 2 for details); higher values describe more curved teeth, and lower values describe straighter teeth.

The degree of lateral compression/flattening was measured as the ratio of the anterior–posterior length of a cross‐section, taken at 50% of the tooth length, to the lingual‐labial length of the cross‐section. To do this, a cross‐section was made through each tooth perpendicular to the midline at 50% of the midline length, and the anterior–posterior and lingual‐labial lengths were measured (see Figure 2 for details). This method is based on a previous protocol from Van Valkenburgh and Ruff (1987). Higher values describe teeth that are more laterally compressed/flattened and ovoid in shape, and lower values describe teeth that are less laterally compressed/flattened and closer to circular in shape. Cross‐sectional shape was also examined qualitatively from the same cross‐section.

## 3 RESULTS

### 3.1 Morphological variation

Saber‐teeth share general characteristics—elongate, slender, flattened—relative to conical toothed taxa. However, considerable morphological variation exists across species and groups, encompassing differences in cusp angle/taper, the degree of lateral compression/flattening, cross‐sectional shape, tip sharpness, curvature, and the presence or absence of serrations. From the sample of saber‐teeth examined in this study, as well as images in the literature, several patterns emerge. Within saber‐tooth taxa, each of the morphological variables (apart from the presence of serrations) exists on a continuum and we observed no distinct groups (e.g., scimitar and dirk tooth (Martin, 1989; Slater and van Valkenburgh, 2008; Figueirido et al., 2018)). Furthermore, we observe overlap between non saber‐tooth/conical tooth taxa and saber‐tooth taxa for all tooth shape variables.

### 3.2 Cusp angle/taper

Saber‐teeth range from highly elongated and slender forms, as seen in species like *Barbourofelis fricki* (aspect ratio = 4.78), *Hoplophoneus primaevus* (aspect ratio = 3.15), and *Smilodon fatalis* (aspect ratio = 3.49), to more robust forms like *Dinofelis piveteaui* (aspect ratio = 1.74) and *Pogonodon platycopis* (aspect ratio = 2.17) (Figure 2). These more robust forms exhibit the same robustness as non saber‐tooth conical forms like the lion (aspect ratio = 1.97). This variation exists on a continuum and does not fall into distinct groups, an observation also supported by recent geometric morphometric studies (Pollock et al., 2025; Shelbourne & Lautenschlager, 2025).

### 3.3 Tip sharpness

Saber‐teeth vary in tip sharpness, ranging from the sharp tips of *B. fricki* (tip surface area (mm^2^) = 0.012), *Thylacosmilus atrox* (tip surface area = 0.014), and *S. fatalis* (tip surface area = 0.015) to the blunter tips of *D. piveteaui* (tip surface area = 0.029) and *P. platycopis* (tip surface area = 0.027) (Figure 2). Values of tip sharpness in the blunter saber‐tooth taxa are similar to those observed in conical non saber‐tooth taxa like the lion (tip surface area = 0.032) and the clouded leopard (tip surface area = 0.021). The conical toothed lion possesses the bluntest tooth tip. The variation observed in tip sharpness exists on a continuum and does not fall into distinct groups. Here, we have endeavored to select teeth that do not show evidence of extreme tooth wear; however, as noted previously, tip sharpness should be interpreted with caution.

### 3.4 Degree of lateral compression/flattening

Saber‐teeth differ in their degree of lateral compression/flattening, ranging from rounder or more circular profiles as seen in *Dinofelis barlowi* (cross‐section ratio = 1.64) and *P. platycopis* (cross‐section ratio = 1.35) to the narrow, laterally compressed forms characteristic of species like *Eusmilus bidentatus* (cross‐section ratio = 2.98) and *Barbourofelis loveorum* (cross‐section ratio = 2.55). The highest degree of lateral compression/flattening of all saber‐tooth taxa is present in *B. fricki* (cross‐section ratio = 4.87) which is almost twice that seen in the next highest value (Figure 2). The degree of lateral compression observed in rounder saber‐tooth forms like *D. barlowi* is similar to that seen in non saber‐tooth forms such as the lion (cross‐section ratio = 1.86) and clouded leopard (cross‐section ratio = 1.43). Like cusp angle/taper, the variation observed for this aspect of tooth shape exists on a continuum and does not fall into distinct groups.

### 3.5 Cross‐sectional shape

Saber‐tooth taxa exhibit a range of cross‐sectional shapes. For example, *Barbourofelis* exhibits a lenticular, figure‐eight‐like cross‐section, while *Smilodon* shows ovoid cross‐sections with pronounced anterior and posterior edges. In contrast, *T. atrox* is triangular in cross‐section (Figure 1), highly unusual for a mammalian canine tooth (T. Pollock personal obvservations). Notably, many saber‐tooth species feature distinct anterior and posterior edges on their canines, a characteristic absent in the conical‐toothed felids in this study, the lion and clouded leopard, which display only posterior edges. Additionally, all saber‐tooth taxa lack the longitudinal lingual (and sometimes labial) indents, known as feline grooves, seen in the lion and clouded leopard that are characteristic of non saber‐tooth felid canines, especially pantherines.

### 3.6 Curvature

Saber‐teeth also show a wide range of variation in tooth curvature, which ranges from relatively straight forms, such as those seen in *D. barlowi* (curvature = 12.83°), *D. piveteaui* (curvature = 6.75°) and *Nimravus brachyops* (curvature = 19.09°), to those with more pronounced curvature, such as *E. bidentatus* (curvature = 51.42°) *T. atrox* (curvature = 49.83°) and *S. fatalis* (curvature = 49.69°) (Figure 2). The degree of curvature observed in straighter saber‐tooth forms like *Dinofelis* is similar to, and sometimes less than, the conical non saber‐tooth taxa like the lion (curvature = 13.41°) and clouded leopard (curvature = 29.08°). The variation observed for this aspect of tooth shape exists on a continuum and does not fall into distinct groups; again, this is supported by recent works (Pollock et al., 2025; Shelbourne & Lautenschlager, 2025).

### 3.7 Serrations

Serrations are another variable feature among saber‐tooth taxa. In some species, such as *S. fatalis*, serrations are present but fine, located exclusively on the canines, and prone to wearing away quickly (Figure 1a; (Whitney et al., 2020)). Others, such as *Homotherium serum* (Figure 1c; Moretti et al., 2025) and *Homotherium latidens* (Serangeli et al., 2015) exhibit more pronounced serrations, which also appear on cheek teeth and deciduous teeth; however, these can wear away quickly. Serrations are also observed in *Amphimachairodus alvarezi* (Figure 1e; Ruiz‐Ramoni et al., 2020), *H. primaevus* (Figure 1b; Barrett, 2016), and *Albanosmilus whitfordi* (Figure 1d; Tseng et al., 2010). Serration morphology itself varies, with species like *S. fatalis* displaying scalloped serrations, while others, such as *H. primaevus* and *A. whitfordi*, have subangular or rounded serrations. Interestingly, serrations appear in both major carnivoran saber‐tooth families—Nimravidae and Felidae (but only in the subfamily Machairodontinae)—whereas species like *Dinofelis* and extant pantherine felids lack serrations entirely (Jiangzuo et al., 2022).

## 4 DISCUSSION

### 4.1 The canine shear‐bite

**FIGURE 3:**
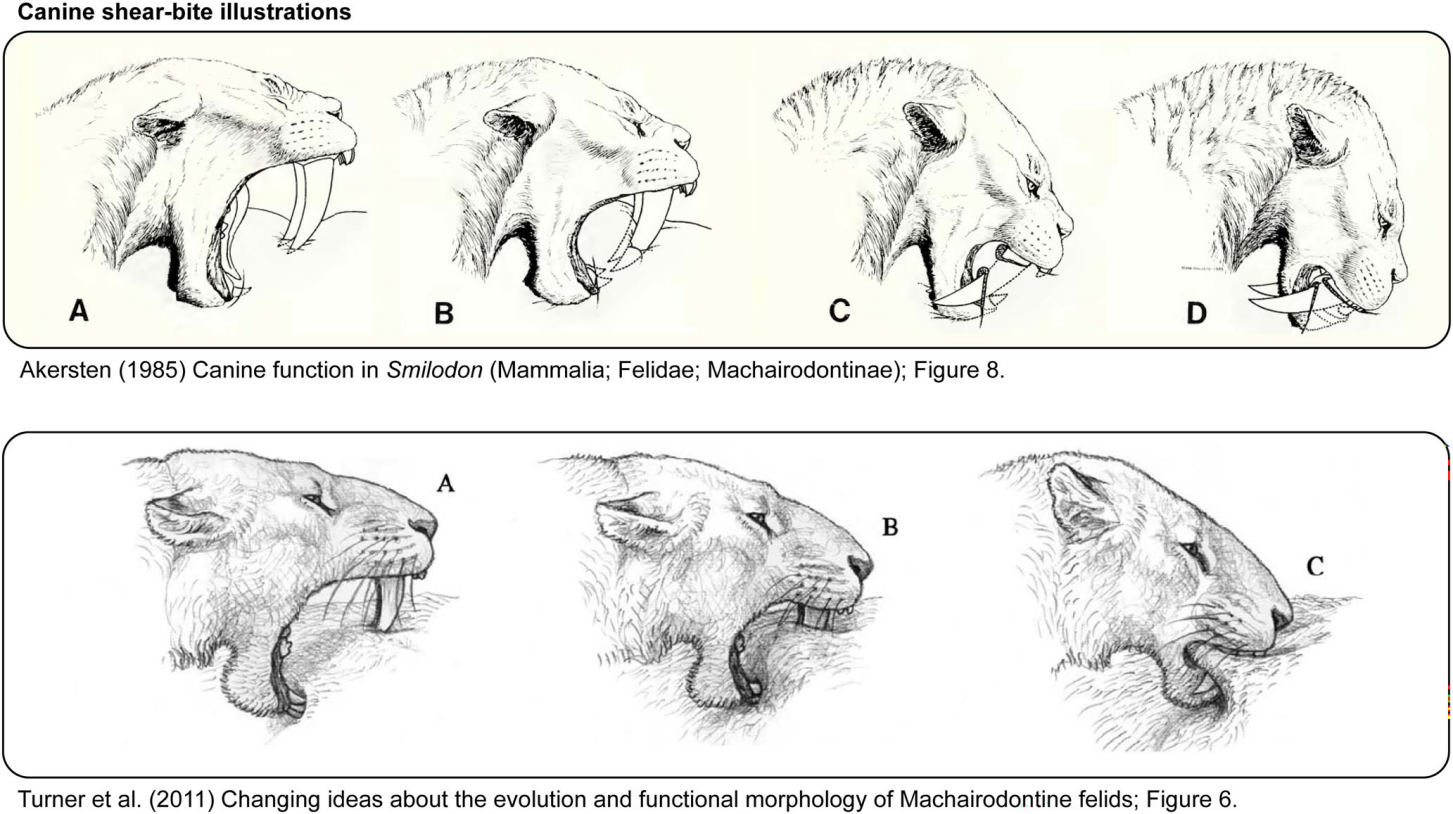
Illustrations of the canine shear‐bite hypothesis from previous studies. The top image is from the original Akersten (1985) article, depicted in fig. 8 of the publication. The bottom image is from Turner et al. (2011), drawn by Mauricio Antón, depicted in fig. 6 of the publication.

Over the years, the canine shear‐bite (or a variation thereof) has emerged as part of the general consensus of how saber‐toothed predators may have killed prey. First coined by Akersten (1985) to describe the killing bite of *Smilodon*, the canine shear‐bite describes a scenario where the elongated upper canines were not used for stabbing or puncturing prey directly, but instead for a shearing action. Here a “shearing action” refers to a biting mechanism where the upper and lower canines work in opposition to create a slicing or shearing effect through tissue. In Akersten (1985) the canine shear bite goes as follows: *Smilodon* presses its open jaws against the prey's body, where the tips of the upper and lower canines create a fold of flesh during initial contact. Here the mandible and lower canines act to anchor the prey, immobilizing its skin (via the fold) and preventing it from moving relative to the bite. Then *Smilodon* uses powerful head depressing muscles to push its head downwards, enabling the upper canines to shear through the prey's flesh in a controlled, slicing motion. In the final stages of the bite, once the incisors are engaged, it is proposed that *Smilodon* could slice through the fold of flesh created by pulling back its head. Akersten (1985) highlights that the fine serrations along the upper canines and the precise alignment of the jaws facilitated a smooth and efficient cutting motion, allowing *Smilodon* to process soft tissues effectively without relying on high bite forces. This proposed killing bite is illustrated nicely in Akersten (1985), fig. 8 and Turner et al. (2011), fig. 6 (both of these illustrations are included in Figure 3 for reference).

The canine shear‐bite contrasts with what we observe today in large non saber/conical‐toothed predators like the lion, which employ a clamp‐and‐hold killing bite (or canine puncture‐bite (Akersten, 1985)). This involves targeting areas like the throat or muzzle, clamping down with a strong bite, and maintaining a prolonged grip to suffocate or immobilize their prey (Ewer, 1968; Sunquist & Sunquist, 2002). When discussing saber‐tooth predatory strategies, these killing bites are often used to represent opposite ends of the spectrum.

To assess the canine shear‐bite, we will examine some of its key elements in light of our current understanding of pointed tooth functional morphology. Here, we will primarily draw on the extreme saber‐tooth forms captured in this study, like *Smilodon*, as well as previous research linking canine morphology to function and biomechanics. However, we note that ours and other recent studies have demonstrated considerable morphological diversity among saber‐tooth forms (Pollock et al., 2025; Shelbourne & Lautenschlager, 2025).

A key, and somewhat obvious, element of the canine shear‐bite hypothesis is that puncture and continued penetration must occur in order to be successful. This contrasts with the clamp‐and‐hold bite where puncture with shallow, or even no, penetration is sufficient to be effective. For example, a lion is still able to hold onto and apply the necessary pressure to compress vital structures of its prey even if its canine teeth do not penetrate deeply into the hide, or in some cases penetrate at all. In contrast, the canine shear‐bite relies on relatively deep (in comparison to the clamp‐and‐hold bite) penetration of the canines into and through the soft tissue. Previous experimental work on tooth wear demonstrated that increasing tooth height and sharpness increased puncture likelihood for pointed teeth in ductile and tough materials (Pollock et al., 2024). In such materials, to initiate a crack a tooth must be pushed deep enough and be sharp enough to concentrate stress at the point of contact and maintain the continued contact required to propagate the crack (Anderson, 2018; Lucas & Luke, 1984). If a tooth is too short or too blunt, neither crack formation nor propagation will occur. The extreme length of extreme saber‐tooth forms, like *Smilodon*, relative to the lion (absolute length: *S. fatalis* = 114.70 mm, *Panthera leo* = 45.20 mm) in combination with sharp tips, slender forms, and bladed edges, all of which decrease the force required to initiate and propagate cracks, suggests a bite that prioritizes puncture with relatively deep penetration, like the canine shear‐bite. This link between tooth morphology and the prioritization of puncture in extreme saber‐tooth forms is supported in a recent study by Pollock et al. (2025), where these teeth were found to be functionally optimized for puncture performance at the expense of breakage resistance.

Another key element of the canine shear‐bite hypothesis is that the biting action goes beyond simple puncture (imagine pushing a pencil into your thigh) to involve puncture combined with some slicing (now imagine pushing a dagger in). Akersten (1985) refers to this as the shearing action. Physical performance tests have shown that the presence of bladed edges along an idealized punch decreases the force required to puncture ductile and tough materials (Freeman & Lemen, 2006). Additionally, mechanical theory predicts that increasing the number of sharp edges will further reduce the force required to penetrate (Freeman, 1992). Sharp edges not only concentrate stresses at the surface of the material but also maintain continued contact with the substrate as the tooth is pushed further in, facilitating puncture with some slicing;the shearing action. As a result, teeth with multiple sharp edges would be expected to perform the canine shear‐bite more effectively. This pattern is consistent with the morphology of many saber‐tooth taxa, which, in contrast to conical‐toothed predators, often exhibit two distinct sharp edges, one along the anterior and one along the posterior of their canines, rather than a single edge restricted to the posterior (Figure 1). Wheeler (2011) tested whether saber‐teeth were capable of producing this shearing action detailed by Akersten (1985) in the famous “robocat” experiments, designed to mimic saber‐tooth biting mechanics. To do this, steel‐edged replicas of *S. fatalis* sabers were attached to a mini track hoe and made to “bite” into large animal carcasses. It was observed that saber‐teeth were capable of producing the shearing action, but only when sufficient up/down motion was incorporated into the bite. Simply driving the canines into the carcass with perpendicular force was insufficient to effectively puncture the hide; instead, successful penetration required a “draw‐cut” motion, where the cutting edges moved parallel to the tooth axis. While some of the experiments carried out by Wheeler (2011) appear to support Akersten's (1985) idea that the shearing component is a key part of a successful saber‐tooth bite, others demonstrate the challenges of achieving sufficient penetration and cutting without highly specific prey conditions or kinematic control. In one notable trial using a male red deer carcass, the teeth followed a shallow trajectory through the belly skin and ultimately lifted the carcass without creating a deep slashing wound, which suggests that the posterior edge of the model teeth failed to breach the hide effectively.

An additional element for the canine shear‐bite hypothesis to consider is the type and direction of forces the teeth are exposed to during biting, which differ from the clamp‐and‐hold bite. In the canine shear‐bite, force is applied vertically, primarily in the anterior–posterior plane, following an arced trajectory dictated by the movement of the skull/neck during the bite. The clamp‐and‐hold bite initially also applies force vertically following the arc of the biting jaws; however, to maintain the grip necessary for suffocating prey, the teeth are also exposed to more variable multidirectional forces (Van Valkenburgh & Ruff, 1987). These differences in the directional forces that teeth are exposed to during biting will favor distinct tooth morphologies, especially in their cross‐sectional geometry. Previous research has demonstrated that canine teeth with laterally compressed cross‐sections are better able to tolerate loads (are stronger) along their anterior–posterior axis, while those with rounder cross‐sections are better able to tolerate multidirectional loads (Christiansen & Wroe, 2007; Jones, 1997; Pollock et al., 2022; Van Valkenburgh & Ruff, 1987). Laterally compressed canines are thought to be associated with behaviors that generate increased stresses along the anterior–posterior axis. For example, canids like the African wild dog (*Lycaon pictus*) have relatively stronger canines in this axis and employ slashing bites to take down prey (Van Valkenburgh & Ruff, 1987). Similarly, Christiansen and Wroe (2007) linked lateral compression of canines to the canine shear‐bite, proposing that this biting behavior would generate significant anterior–posterior stresses in the canines, favoring increased bending strength in this plane.

If we look specifically at the arced trajectory of the tooth expected during the canine shear‐bite, the increased curvature observed in extreme saber‐tooth forms is also likely favorable. Higher curvature would allow the tooth to follow such a path during penetration, facilitating its movement through and out of the prey relative to a straighter tooth. Additionally, this curvature helps maintain the tip of the tooth in a perpendicular orientation to the prey during biting, which reduces the stresses experienced by the tooth tip (Pollock et al., 2022; Pollock et al., 2025; Zhang et al., 2024). If saber‐teeth were straight, we would expect the canine shear‐bite to be less mechanically viable, perhaps not possible.

While the morphological traits of extreme saber‐tooth forms appear more biomechanically compatible with the canine shear‐bite than the clamp‐and‐hold bite, the model also presents several limitations. First, the canine shear‐bite relies on a shallow angle of entry and a specific arc of motion to achieve effective slicing, conditions that may only occur when targeting compliant, convex soft tissues. Andersson et al. (2011) noted that these surface geometries are relatively rare, particularly in large prey, and that shallow bites are more likely to fail on flatter surfaces. Second, the bite requires substantial head depression and, potentially, powerful forelimbs to immobilize the prey during the controlled draw‐cut motion. This combination of requirements may limit the feasibility of the canine shear‐bite in taxa lacking robust forelimb anatomy, such as *Homotherium*. These biomechanical constraints highlight that, while the canine shear‐bite may have been effective in specific contexts, it may represent one strategy within a broader spectrum of saber‐tooth functional diversity.

## 5 CONCLUSION

By considering the unique morphology of extreme saber‐tooth forms—elongate, slender, sharp tips and edges, curved, and laterally compressed—along with our current understanding of puncture mechanics and pointed tooth functional morphology, extreme saber‐tooth forms appear better suited to a canine shear‐like bite rather than a clamp‐and‐hold bite. However, we (and others (Pollock et al., 2025; Shelbourne & Lautenschlager, 2025)) have demonstrated that there is considerable morphological diversity among saber‐tooth forms and this appears along a continuum from the shorter, more robust, and straighter but still sharp‐tipped and edged teeth we find in *Dinofelis* and *Nimravus* to the extreme forms we find in *Hopolophoneus*, *Smilodon*, and *Barbourofelis*. This suggests greater functional diversity beyond the canine shear‐bite vs. clamp‐and‐hold bite dichotomy, which is a growing consensus in recent research (Chatar et al., 2022, 2024; Figueirido et al., 2018; Figueirido et al., 2025; Lautenschlager et al., 2020; Pollock et al., 2025; Shelbourne & Lautenschlager, 2025).

There is still much we do not know about saber teeth or pointed tooth biomechanics in general. In particular, key gaps remain in our understanding of how curvature and cross‐sectional geometry influence puncture mechanics. While previous studies have demonstrated that cross‐sectional shape impacts bending strength and directional resistance, most of this work has been based on simple models—either stretching or squashing a circle or testing basic indenter designs (Freeman & Lemen, 2006). Yet, saber‐tooth taxa exhibit a broad range of cross‐sectional geometries, from the figure‐eight profile of *Barbourofelis* to the triangular shape of *Thylacosmilus*, raising questions about how these different forms affect puncture mechanics and performance. Similarly, the role of serrations in puncture and cutting remains largely unexplored, despite experimental work showing that serrated edges enhance grip and reduce penetration forces. These open questions are now more accessible to investigation thanks to emerging methodologies. Approaches such as digital image correlation, dynamic finite element analysis, and particle tracking velocimetry allow for more detailed biomechanical testing of pointed teeth in realistic loading conditions, which although in their infancy in terms of tooth research, are highly promising (Zhang et al., 2024; Anderson et al., 2019). Additionally, optimisation studies, like those conducted in our own research, offer a powerful framework for understanding the functional constraints and evolutionary pathways of saber teeth (Pollock et al., 2025), or more broadly pointed teeth in general. There is still a lot to uncover, but the future of tooth biomechanics is promising.

## AUTHOR CONTRIBUTIONS

**Tahlia Pollock:** Conceptualization; data curation; formal analysis; investigation; methodology; visualization; writing – original draft; writing – review and editing. **Philip S. L. Anderson:** Conceptualization; supervision; writing – original draft; writing – review and editing.

## FUNDING INFORMATION

T. Pollock was supported by a John Templeton Foundation Grant (JTF 62574) (awarded to Emily J. Rayfield. and Philip C.J. Donoghue) while writing this article. The opinions expressed in this article are those of the author and do not necessarily reflect the views of the John Templeton Foundation). P. Anderson's contributions to this work were supported by the National Science Foundation (grant NSF IOS 19‐42906 CAR to P.S.L.A).

## CONFLICT OF INTEREST STATEMENT

Tahlia Pollock is one of the editors for this saber‐tooth Special Issue.

## Supporting information


**Data S1:** Supporting Information.

## Data Availability

The original 3D surface file data of canine tooth models from saber‐toothed taxa have been deposited at MorphoSource and are publicly available as of the date of publication in the project “Functional optimality drives the repeated evolution of extreme saber‐tooth forms” (https://www.morphosource.org/projects/000699335) and the conical toothed taxa are available in the project “Canine teeth of mammalian carnivores: the killer's toolkit” (https://www.morphosource.org/projects/000345195). Reconstructed tooth models, for saber‐teeth, will be shared by T. Pollock upon request.

## References

[ar25690-bib-0001] Abler, W. L. (1992). The serrated teeth of tyrannosaurid dinosaurs, and biting structures in other animals. Paleobiology, 18, 161–183.

[ar25690-bib-0002] Akersten, W. (1985). Canine function in *Smilodon* (Mammalia; Felidae; Machairodontinae). Los Angeles Contributions in Science, 356, 1–22.

[ar25690-bib-0003] Anderson, P. S. (2009). The effects of trapping and blade angle of notched dentitions on fracture of biological tissues. Journal of Experimental Biology, 212(Pt 22), 3627–3632. 10.1242/jeb.033712 19880723

[ar25690-bib-0004] Anderson, P. S. (2018). Making a point: Shared mechanics underlying the diversity of biological puncture. Journal of Experimental Biology, 221, jeb187294. 30446527 10.1242/jeb.187294

[ar25690-bib-0005] Anderson, P. S. , Crofts, S. B. , Kim, J.‐T. , & Chamorro, L. P. (2019). Taking a stab at quantifying the energetics of biological puncture. Integrative and Comparative Biology, 59, 1586–1596. 31141122 10.1093/icb/icz078

[ar25690-bib-0006] Anderson, P. S. , Gill, P. G. , & Rayfield, E. J. (2011). Modeling the effects of cingula structure on strain patterns and potential fracture in tooth enamel. Journal of Morphology, 272, 50–65. 20960463 10.1002/jmor.10896

[ar25690-bib-0007] Anderson, P. S. , & LaBarbera, M. (2008). Functional consequences of tooth design: Effects of blade shape on energetics of cutting. Journal of Experimental Biology, 211(Pt 22), 3619–3626. 10.1242/jeb.020586 18978227

[ar25690-bib-0008] Anderson, P. S. L. , LaCosse, J. , & Pankow, M. (2016). Point of impact: The effect of size and speed on puncture mechanics. Interface Focus, 6, 20150111. 27274801 10.1098/rsfs.2015.0111 PMC4843624

[ar25690-bib-0009] Andersson, K. , Norman, D. , & Werdelin, L. (2011). Sabretoothed carnivores and the killing of large prey. PLoS One, 6, e24971. 22039403 10.1371/journal.pone.0024971 PMC3198467

[ar25690-bib-0010] Antón, M. , & Galobart, À. (1999). Neck function and predatory behavior in the scimitar toothed cat *Homotherium latidens* (Owen). Journal of Vertebrate Paleontology, 19, 771–784.

[ar25690-bib-0011] Antón, M. , Salesa, M. J. , Pastor, J. F. , Sanchez, I. M. , Fraile, S. , & Morales, J. (2004). Implications of the mastoid anatomy of larger extant felids for the evolution and predatory behaviour of sabretoothed cats (Mammalia, Carnivora, Felidae). Zoological Journal of the Linnean Society, 140, 207–221.

[ar25690-bib-0012] Ballell, A. , Benton, M. J. , & Rayfield, E. J. (2022). Dental form and function in the early feeding diversification of dinosaurs. Science Advances, 8, eabq5201. 36525501 10.1126/sciadv.abq5201 PMC9757754

[ar25690-bib-0013] Ballell, A. , & Ferrón, H. G. (2021). Biomechanical insights into the dentition of megatooth sharks (Lamniformes: Otodontidae). Scientific Reports, 11, 1–9. 33441828 10.1038/s41598-020-80323-z PMC7806677

[ar25690-bib-0014] Barrett, P. Z. (2016). Taxonomic and systematic revisions to the north American Nimravidae (Mammalia, Carnivora). PeerJ, 4, e1658. 26893959 10.7717/peerj.1658 PMC4756750

[ar25690-bib-0015] Berthaume, M. , Grosse, I. R. , Patel, N. D. , Strait, D. S. , Wood, S. , & Richmond, B. G. (2010). The effect of early hominin occlusal morphology on the fracturing of hard food items. The Anatomical Record: Advances in Integrative Anatomy and Evolutionary Biology, 293, 594–606. 20235316 10.1002/ar.21130

[ar25690-bib-0016] Biknevicius, A. , van Valkenburgh, B. , & Walker, J. (1996). Incisor size and shape: Implications for feeding behaviors in saber‐toothed “cats”. Journal of Vertebrate Paleontology, 16, 510–521.

[ar25690-bib-0017] Bocherens, H. , Cotte, M. , Bonini, R. , Scian, D. , Straccia, P. , Soibelzon, L. , & Prevosti, F. J. (2016). Paleobiology of sabretooth cat *Smilodon* populator in the Pampean region (Buenos Aires Province, Argentina) around the last glacial maximum: Insights from carbon and nitrogen stable isotopes in bone collagen. Palaeogeography, Palaeoclimatology, Palaeoecology, 449, 463–474.

[ar25690-bib-0018] Chatar, N. , Fischer, V. , Siliceo, G. , Antón, M. , Morales, J. , & Salesa, M. J. (2021). Morphometric analysis of the mandible of primitive Sabertoothed felids from the late Miocene of Spain. Journal of Mammalian Evolution, 28, 753–771.

[ar25690-bib-0019] Chatar, N. , Fischer, V. , & Tseng, Z. J. (2022). Many‐to‐one function of cat‐like mandibles highlights a continuum of sabre‐tooth adaptations. Proceedings of the Royal Society B, 289, 20221627. 36475442 10.1098/rspb.2022.1627 PMC9727663

[ar25690-bib-0020] Chatar, N. , Michaud, M. , Tamagnini, D. , & Fischer, V. (2024). Evolutionary patterns of cat‐like carnivorans unveil drivers of the sabertooth morphology. Current Biology, 34, 2460–2473.e4. 38759651 10.1016/j.cub.2024.04.055

[ar25690-bib-0021] Cho, W. K. , Ankrum, J. A. , Guo, D. , Chester, S. A. , Yang, S. Y. , Kashyap, A. , Campbell, G. A. , Wood, R. J. , Rijal, R. K. , & Karnik, R. (2012). Microstructured barbs on the north American porcupine quill enable easy tissue penetration and difficult removal. Proceedings of the National Academy of Sciences, 109, 21289–21294. 10.1073/pnas.1216441109 PMC3535670 23236138

[ar25690-bib-0022] Christiansen, P. (2007). Comparative bite forces and canine bending strength in feline and sabretooth felids: Implications for predatory ecology. Zoological Journal of the Linnean Society, 151, 423–437.

[ar25690-bib-0023] Christiansen, P. (2008). Evolutionary convergence of primitive sabertooth craniomandibular morphology: The clouded leopard (Neofelis nebulosa) and *Paramachairodus ogygia* compared. Journal of Mammalian Evolution, 15, 155–179.

[ar25690-bib-0024] Christiansen, P. (2011). A dynamic model for the evolution of sabrecat predatory bite mechanics. Zoological Journal of the Linnean Society, 162, 220–242.

[ar25690-bib-0025] Christiansen, P. , & Wroe, S. (2007). Bite forces and evolutionary adaptations to feeding ecology in carnivores. Ecology, 88, 347–358. 17479753 10.1890/0012-9658(2007)88[347:bfaeat]2.0.co;2

[ar25690-bib-0026] Cleuren, S. G. , Hocking, D. P. , & Evans, A. R. (2021). Fang evolution in venomous snakes: Adaptation of 3D tooth shape to the biomechanical properties of their prey. Evolution, 75, 1377–1394. 33904594 10.1111/evo.14239

[ar25690-bib-0027] Corn, K. A. , Farina, S. C. , Brash, J. , & Summers, A. P. (2016). Modelling tooth–prey interactions in sharks: The importance of dynamic testing. Royal Society Open Science, 3, 160141. 27853592 10.1098/rsos.160141 PMC5108942

[ar25690-bib-0028] Crofts, S. , Lai, Y. , Hu, Y. , & Anderson, P. (2019). How do morphological sharpness measures relate to puncture performance in viperid snake fangs? Biology Letters, 15, 20180905. 30991915 10.1098/rsbl.2018.0905 PMC6501362

[ar25690-bib-0029] Crofts, S. , Smith, S. , & Anderson, P. (2020). Beyond description: The many facets of dental biomechanics. Integrative and Comparative Biology, 60, 594–607. 32652006 10.1093/icb/icaa103

[ar25690-bib-0030] Crofts, S. , & Summers, A. (2014). How to best smash a snail: The effect of tooth shape on crushing load. Journal of the Royal Society Interface, 11, 20131053. 24430124 10.1098/rsif.2013.1053 PMC3899874

[ar25690-bib-0031] Crofts, S. B. , & Anderson, P. S. L. (2018). The influence of cactus spine surface structure on puncture performance and anchoring ability is tuned for ecology. Proceedings of the Royal Society B: Biological Sciences, 285, 20182280. 10.1098/rspb.2018.2280 PMC6253362 30464068

[ar25690-bib-0032] Crofts, S. B. , & Anderson, P. S. L. (2023). How venom pore placement may influence puncture performance in snake fangs. Journal of Experimental Biology, 226(17), jeb245666. 10.1242/jeb.245666 37642375

[ar25690-bib-0033] DeSantis, L. R. (2016). Dental microwear textures: Reconstructing diets of fossil mammals. Surface Topography: Metrology and Properties, 4, 023002.

[ar25690-bib-0034] DeSantis, L. R. , Schubert, B. W. , Scott, J. R. , & Ungar, P. S. (2012). Implications of diet for the extinction of saber‐toothed cats and American lions. PLoS One, 7, e52453. 23300674 10.1371/journal.pone.0052453 PMC3530457

[ar25690-bib-0035] Deutsch, A. R. , Berger, A. , Martens, L. L. , Witt, B. R. , Smith, R. L. J. , & Hartstone‐Rose, A. (2025). Myological and osteological approaches to gape and bite force reconstruction in *Smilodon fatalis* . The Anatomical Record. 10.1002/ar.25529 PMC12506842 38943271

[ar25690-bib-0036] Duckler, G. L. (1997). Parietal depressions in skulls of the extinct saber‐toothed felid *Smilodon fatalis*: Evidence of mechanical strain. Journal of Vertebrate Paleontology, 17(3), 600–609. 10.1080/02724634.1997.10011006

[ar25690-bib-0037] Dumont, E. , Grosse, I. R. , & Slater, G. J. (2009). Requirements for comparing the performance of finite element models of biological structures. Journal of Theoretical Biology, 256, 96–103. 18834892 10.1016/j.jtbi.2008.08.017

[ar25690-bib-0038] Dumont, E. R. , Piccirillo, J. , & Grosse, I. R. (2005). Finite‐element analysis of biting behavior and bone stress in the facial skeletons of bats. The Anatomical Record Part A, Discoveries in Molecular, Cellular, and Evolutionary Biology, 283(2), 319–330. 10.1002/ar.a.20165 15747350

[ar25690-bib-0039] Emerson, S. B. , & Radinsky, L. (1980). Functional analysis of sabertooth cranial morphology. Paleobiology, 6, 295–312.

[ar25690-bib-0040] Evans, A. , & Sanson, G. (1998). The effect of tooth shape on the breakdown of insects. Journal of Zoology, 246, 391–400.

[ar25690-bib-0041] Evans, A. R. , & Pineda‐Munoz, S. (2018). Inferring mammal dietary ecology from dental morphology. In D. A. Croft , D. F. Su , & S. W. Simpson (Eds.), Methods in paleoecology: Reconstructing Cenozoic terrestrial environments and ecological communities (pp. 37–51). Springer International Publishing.

[ar25690-bib-0042] Evans, A. R. , Pollock, T. I. , Cleuren, S. G. , Parker, W. M. , Richards, H. L. , Garland, K. L. , Fitzgerald, E. M. , Wilson, T. E. , Hocking, D. P. , & Adams, J. W. (2021). A universal power law for modelling the growth and form of teeth, claws, horns, thorns, beaks, and shells. BMC Biology, 19, 1–14. 33781258 10.1186/s12915-021-00990-w PMC8008625

[ar25690-bib-0043] Evans, A. R. , & Sanson, G. D. (2003). The tooth of perfection: Functional and spatial constraints on mammalian tooth shape. Biological Journal of the Linnean Society, 78, 173–191.

[ar25690-bib-0127] Evans, A. R ., Hunter, J. , Fortelius, M. , & Sanson, G. D . (2005). The scaling of tooth sharpness in mammals. In Annales Zoologici Fennici (pp. 603–613). Finnish Zoological and Botanical Publishing Board.

[ar25690-bib-0044] Evans, A. R. , Wilson, G. P. , Fortelius, M. , & Jernvall, J. (2007). High‐level similarity of dentitions in carnivorans and rodents. Nature, 445, 78–81. 17167416 10.1038/nature05433

[ar25690-bib-0045] Ewer, R. (1973). The carnivores. Cornell University Press.

[ar25690-bib-0046] Ewer, R. F. (1968). Ethology of mammals. Logos Press Limited.

[ar25690-bib-0047] Figueirido, B. , Lautenschlager, S. , Pérez‐Ramos, A. , & van Valkenburgh, B. (2018). Distinct predatory behaviors in scimitar‐and dirk‐toothed sabertooth cats. Current Biology, 28, 3260–3266.e3. 30293717 10.1016/j.cub.2018.08.012

[ar25690-bib-0048] Figueirido, B. , Tucker, S. , & Lautenschlager, S. (2025). Comparing cranial biomechanics between *Barbourofelis fricki* and *Smilodon fatalis*: Is there a universal killing‐bite among saber‐toothed predators? The Anatomical Record. 10.1002/ar.25451 PMC12506856 38613218

[ar25690-bib-0049] Fischer, V. , Bennion, R. F. , Foffa, D. , MacLaren, J. A. , McCurry, M. R. , Melstrom, K. M. , & Bardet, N. (2022). Ecological signal in the size and shape of marine amniote teeth. Proceedings of the Royal Society B, Biological Sciences, 289, 20221214. 10.1098/rspb.2022.1214 PMC9470252 36100016

[ar25690-bib-0050] Frazzetta, T. (1988). The mechanics of cutting and the form of shark teeth (Chondrichthyes, Elasmobranchii). Zoomorphology, 108, 93–107.

[ar25690-bib-0051] Frazzetta, T. H. (1966). Studies on the morphology and function of the skull in the Boidae (Serpentes). Part II. Morphology and function of the jaw apparatus in *Python sebae* and *Python molurus* . Journal of Morphology, 118(2), 217–295. 10.1002/jmor.1051180206 5948607

[ar25690-bib-0052] Freeman, P. W. (1992). Canine teeth of bats (Microchiroptera): Size, shape and role in crack propagation. Biological Journal of the Linnean Society, 45, 97–115.

[ar25690-bib-0053] Freeman, P. W. , & Lemen, C. (2006). Puncturing ability of idealized canine teeth: Edged and non‐edged shanks. Journal of Zoology, 269, 51–56.

[ar25690-bib-0054] Freeman, P. W. , & Lemen, C. (2007a). An experimental approach to modeling the strength of canine teeth. Journal of Zoology, 271, 162–169.

[ar25690-bib-0055] Freeman, P. W. , & Lemen, C. A. (2007b). The trade‐off between tooth strength and tooth penetration: Predicting optimal shape of canine teeth. Journal of Zoology, 273, 273–280.

[ar25690-bib-0056] Freeman, P. W. , & Weins, W. N. (1997). Puncturing ability of bat canine teeth: The tip. Mammalogy Papers (p. 9). University of Nebraska State Museum.

[ar25690-bib-0057] Goldschmidt, S. , Zimmerman, C. , Collins, C. , Hetzel, S. , Ploeg, H.‐L. , & Soukup, J. W. (2017). The influence of force direction on the fracture pattern and fracture resistance of canine teeth in dogs. Journal of Veterinary Dentistry, 34, 8–17. 28446072 10.1177/0898756417705229

[ar25690-bib-0058] Hartstone‐Rose, A. , & Wahl, S. (2008). Using radii‐of‐curvature for the reconstruction of extinct south African carnivoran masticatory behavior. Comptes Rendus Palevol, 7, 629–643.

[ar25690-bib-0059] Hendrickx, C. , Mateus, O. , & Araújo, R. (2015). A proposed terminology of theropod teeth (Dinosauria, Saurischia). Journal of Vertebrate Paleontology, 35, e982797.

[ar25690-bib-0060] Janis, C. M. , Figueirido, B. , DeSantis, L. , & Lautenschlager, S. (2020). An eye for a tooth: *Thylacosmilus* was not a marsupial “saber‐tooth predator”. PeerJ, 8, e9346. 32617190 10.7717/peerj.9346 PMC7323715

[ar25690-bib-0061] Jiangzuo, Q. , Li, S. , Fu, J. , Wang, S. , Ji, X. , Duan, M. , & Che, D. (2022). Fossil Felidae (Carnivora: Mammalia) from the Yuanmou hominid site, southern China (late Miocene) and its significance in the living environment of the fossil ape. Zoological Journal of the Linnean Society, 196, 1156–1174.

[ar25690-bib-0062] Jones, D. , Evans, A. R. , Siu, K. K. , Rayfield, E. J. , & Donoghue, P. C. (2012). The sharpest tools in the box? Quantitative analysis of conodont element functional morphology. Proceedings of the Royal Society B: Biological Sciences, 279, 2849–2854. 10.1098/rspb.2012.0147 PMC3367778 22418253

[ar25690-bib-0063] Jones, M. (1997). Character displacement in Australian dasyurid carnivores: Size relationships and prey size patterns. Ecology, 78, 2569–2587.

[ar25690-bib-0064] Kajiura, S. M. , & Tricas, T. C. (1996). Seasonal dynamics of dental sexual dimorphism in the Atlantic stingray *Dasyatis sabina* . Journal of Experimental Biology, 199, 2297–2306. 9320215 10.1242/jeb.199.10.2297

[ar25690-bib-0065] Kardong, K. V. (1980). Evolutionary patterns in advanced snakes. American Zoologist, 20, 269–282.

[ar25690-bib-0066] King, J. E. (1983). Seals of the world. British Museum (Natural History).

[ar25690-bib-0067] Lautenschlager, S. , Figueirido, B. , Cashmore, D. D. , Bendel, E.‐M. , & Stubbs, T. L. (2020). Morphological convergence obscures functional diversity in sabre‐toothed carnivores. Proceedings of the Royal Society B, 287, 20201818. 32993469 10.1098/rspb.2020.1818 PMC7542828

[ar25690-bib-0068] Lucas, P. (1982). Basic principles of tooth design. In Teeth: Form, function and evolution (pp. 154–162). Columbia University Press.

[ar25690-bib-0069] Lucas, P. , & Luke, D. (1984). Chewing it over: Basic principles of food breakdown. In Food acquisition and processing in primates (pp. 283–301). Springer Sciences.

[ar25690-bib-0070] Lucas, P. W. (2004). Dental functional morphology: How teeth work. Cambridge University Press.

[ar25690-bib-0071] MacKenzie, A. S. , Brock, G. A. , & McCurry, M. R. (2024). The impact of apicobasal ridges on dental load‐bearing capacity in aquatic‐feeding predatory amniotes. Paleobiology, 50(2), 346–363. 10.1017/pab.2024.10

[ar25690-bib-0072] Martin, L. , Babiarz, J. , Naples, V. , & Hearst, J. (2000). Three ways to be a saber‐toothed cat. Naturwissenschaften, 87, 41–44. 10663132 10.1007/s001140050007

[ar25690-bib-0073] Martin, L. D. (1989). Fossil history of the terrestrial Carnivora. In Carnivore behavior, ecology, and evolution (pp. 536–568). Springer.

[ar25690-bib-0074] McCurry, M. R. , Evans, A. R. , Fitzgerald, E. M. G. , McHenry, C. R. , Bevitt, J. , & Pyenson, N. D. (2019). The repeated evolution of dental apicobasal ridges in aquatic‐feeding mammals and reptiles. Biological Journal of the Linnean Society, 127, 245–259.

[ar25690-bib-0075] McHenry, C. R. , Wroe, S. , Clausen, P. D. , Moreno, K. , & Cunningham, E. (2007). Supermodeled sabercat, predatory behavior in *Smilodon fatalis* revealed by high‐resolution 3D computer simulation. Proceedings of the National Academy of Sciences of the United States of America, 104, 16010–16015. 17911253 10.1073/pnas.0706086104 PMC2042153

[ar25690-bib-0076] Meachen‐Samuels, J. A. , & van Valkenburgh, B. (2010). Radiographs reveal exceptional forelimb strength in the sabertooth cat, *Smilodon fatalis* . PLoS One, 5(7), e11412. 10.1371/journal.pone.0011412 20625398 PMC2896400

[ar25690-bib-0077] Melchionna, M. , Profico, A. , Castiglione, S. , Serio, C. , Mondanaro, A. , Modafferi, M. , Tamagnini, D. , Maiorano, L. , Raia, P. , & Witmer, L. M. (2021). A method for mapping morphological convergence on three‐dimensional digital models: The case of the mammalian sabre‐tooth. Palaeontology, 64(4), 573–584. 10.1111/pala.12542

[ar25690-bib-0078] Moretti, J. A. , Flores, D. , Bell, C. J. , Godwin, W. , Hartstone‐Rose, A. , & Lewis, P. J. (2025). The scimitar‐cat *Homotherium* from the submerged continental shelf of the Gulf coast of Texas. The Anatomical Record. 10.1002/ar.25461 38654480

[ar25690-bib-0079] Moyer, J. K. , & Bemis, W. E. (2017). Shark teeth as edged weapons: Serrated teeth of three species of selachians. Zoology, 120, 101–109. 27353190 10.1016/j.zool.2016.05.007

[ar25690-bib-0080] Palci, A. , Lee, M. S. Y. , Crowe‐Riddell, J. M. , & Sherratt, E. (2023). Shape and size variation in elapid Snake fangs and the effects of phylogeny and diet. Evolutionary Biology, 50, 476–487.

[ar25690-bib-0128] Pardo‐Judd J , & DeSantis, L. (2025). Dietary ecology of Smilodon across time and space: Additional perspectives from Smilodon gracilis and Smilodon fatalis in Florida. The Anatomical Record. 10.1002/ar.25648 PMC12506855 40181504

[ar25690-bib-0081] Piras, P. , Silvestro, D. , Carotenuto, F. , Castiglione, S. , Kotsakis, A. , Maiorino, L. , Melchionna, M. , Mondanaro, A. , Sansalone, G. , & Serio, C. (2018). Evolution of the sabertooth mandible: A deadly ecomorphological specialization. Palaeogeography, Palaeoclimatology, Palaeoecology, 496, 166–174.

[ar25690-bib-0082] Pollock, T. I. , Deakin, W. J. , Chatar, N. , Milla Carmona, P. S. , Rovinsky, D. S. , Panagiotopoulou, O. , Parker, W. M. G. , Adams, J. W. , Hocking, D. P. , Donoghue, P. C. J. , Rayfield, E. J. , & Evans, A. R. (2025). Functional optimality underpins the repeated evolution of the extreme “saber‐tooth” morphology. Current Biology, 35, 455–467.e6. 39793568 10.1016/j.cub.2024.11.059

[ar25690-bib-0083] Pollock, T. I. , Hocking, D. P. , & Evans, A. R. (2022). The killer's toolkit: Remarkable adaptations in the canine teeth of mammalian carnivores. Zoological Journal of the Linnean Society, 196(3), 1138–1155.

[ar25690-bib-0084] Pollock, T. I. , Hocking, D. P. , & Evans, A. R. (2024). Is a blunt sword pointless? Tooth wear impacts puncture performance in Tasmanian devil canines. Journal of Experimental Biology, 227(3), jeb246925. 10.1242/jeb.246925 38099427 PMC10917061

[ar25690-bib-0085] Pollock, T. I. , Panagiotopoulou, O. , Hocking, D. P. , & Evans, A. R. (2022). Taking a stab at modelling canine tooth biomechanics in mammalian carnivores with beam theory and finite‐element analysis. Royal Society Open Science, 9, 220701. 36300139 10.1098/rsos.220701 PMC9579775

[ar25690-bib-0086] Popowics, T. E. , & Fortelius, M. (1997). On the cutting edge: Tooth blade sharpness in herbivorous and faunivorous mammals. Annales Zoologici Fennici, 34, 73–88.

[ar25690-bib-0087] Quan, H. , Liang, X. , Zhang, X. , Meyers, M. A. , McMeeking, R. M. , & Arzt, E. (2024). The shape of Nature's stingers revealed. Proceedings of the National Academy of Sciences of the United States of America, 121, e2316320121. 38319966 10.1073/pnas.2316320121 PMC10873632

[ar25690-bib-0088] Rajabizadeh, M. , van Wassenbergh, S. , Mallet, C. , Rücklin, M. , & Herrel, A. (2021). Tooth‐shape adaptations in aglyphous colubrid snakes inferred from three‐dimensional geometric morphometrics and finite element analysis. Zoological Journal of the Linnean Society, 191, 454–467.

[ar25690-bib-0089] Rayfield, E. J. (2007). Finite element analysis and understanding the biomechanics and evolution of living and fossil organisms. Annual Review of Earth and Planetary Sciences, 35, 541–576.

[ar25690-bib-0090] Reidman, M. (1990). The pinnipeds: Seals, sea lions and walruses. University of California Press.

[ar25690-bib-0091] Richmond, B. G. , Wright, B. W. , Grosse, I. , Dechow, P. C. , Ross, C. F. , Spencer, M. A. , & Strait, D. S. (2005). Finite element analysis in functional morphology. The Anatomical Record Part A, Discoveries in Molecular, Cellular, and Evolutionary Biology, 283, 259–274. 15747355 10.1002/ar.a.20169

[ar25690-bib-0092] Ruiz‐Ramoni, D. , Montellano‐Ballesteros, M. , Rincón, A. D. , Solórzano, A. , & Guzmán, G. (2020). Presence of *Amphimachairodus coloradensis* (Cook, 1922) (Felidae: Machairodontinae) in the Neogene of Hidalgo, Central Mexico. Journal of South American Earth Sciences, 100, 102550.

[ar25690-bib-0093] Schofield, R. M. S. , Choi, S. , Coon, J. J. , Goggans, M. S. , Kreisman, T. F. , Silver, D. M. , & Nesson, M. H. (2016). Is fracture a bigger problem for smaller animals? Force and fracture scaling for a simple model of cutting, puncture and crushing. Interface Focus, 6, 20160002. 27274804 10.1098/rsfs.2016.0002 PMC4843627

[ar25690-bib-0094] Segall, M. , Houssin, C. , Delapré, A. , Cornette, R. , Herrel, A. , Milgram, J. , Shahar, R. , & Dumont, M. (2023). Armed to the teeth: The underestimated diversity in tooth shape in snakes and its relation to feeding behavior and diet. Ecology and Evolution, 13, e10011. 37066060 10.1002/ece3.10011 PMC10099486

[ar25690-bib-0095] Serangeli, J. , van Kolfschoten, T. , Starkovich, B. M. , & Verheijen, I. (2015). The European saber‐toothed cat (*Homotherium latidens*) found in the “spear horizon” at Schöningen (Germany). Journal of Human Evolution, 89, 172–180. 26505304 10.1016/j.jhevol.2015.08.005

[ar25690-bib-0096] Shelbourne, C. D. , & Lautenschlager, S. (2025). Morphological diversity of saber‐tooth upper canines and its functional implications. The Anatomical Record. 10.1002/ar.25458 PMC12506846 38646928

[ar25690-bib-0097] Shergold, O. A. , & Fleck, N. A. (2005). Experimental investigation into the deep penetration of soft solids by sharp and blunt punches, with application to the piercing of skin. Journal of Biomechanical Engineering, 127, 838–848. 16248314 10.1115/1.1992528

[ar25690-bib-0098] Simpson, G. G. (1941). The function of saber‐like canines in carnivorous mammals. American Museum of Natural History.

[ar25690-bib-0099] Skamniotis, C. (2017). Characterising and modelling fracture in functional pet foods [PhD Thesis]. Imperial College London.

[ar25690-bib-0100] Skamniotis, C. , Elliott, M. , & Charalambides, M. (2017). On modeling the large strain fracture behaviour of soft viscous foods. Physics of Fluids, 29, 121610.

[ar25690-bib-0101] Slater, G. J. , & van Valkenburgh, B. (2008). Long in the tooth: Evolution of sabertooth cat cranial shape. Paleobiology, 34, 403–419.

[ar25690-bib-0102] Soukup, J. W. , Collins, C. , & Ploeg, H.‐L. (2015). The influence of crown height to diameter ratio on the force to fracture of canine teeth in dogs. Journal of Veterinary Dentistry, 32, 155–163. 26638294 10.1177/089875641503200302 PMC5140095

[ar25690-bib-0103] Strait, S. G. (1993). Molar morphology and food texture among small‐bodied insectivorous mammals. Journal of Mammalogy, 74, 391–402.

[ar25690-bib-0104] Sunquist, M. , & Sunquist, F. (2002). Wild cats of the world. University of Chicago Press.

[ar25690-bib-0105] Therrien, F. (2005). Feeding behaviour and bite force of sabretoothed predators. Zoological Journal of the Linnean Society, 145, 393–426.

[ar25690-bib-0106] Torices, A. , Wilkinson, R. , Arbour, V. M. , Ruiz‐Omeñaca, J. I. , & Currie, P. J. (2018). Puncture‐and‐pull biomechanics in the teeth of predatory Coelurosaurian dinosaurs. Current Biology, 28, 1467–1474. 29706515 10.1016/j.cub.2018.03.042

[ar25690-bib-0107] Tseng, Z. J. (2025). Bending performance changes during prolonged canine eruption in saber‐toothed carnivores: A case study of *Smilodon fatalis* . The Anatomical Record. 10.1002/ar.25447 PMC12506851 38588019

[ar25690-bib-0108] Tseng, Z. J. , Takeuchi, G. T. , & Wang, X. (2010). Discovery of the upper dentition of *Barbourofelis whitfordi* (Nimravidae, Carnivora) and an evaluation of the genus in California. Journal of Vertebrate Paleontology, 30, 244–254.

[ar25690-bib-0109] Turner, A. , Antón, M. , Salesa, M. , & Morales, J. (2011). Changing ideas about the evolution and functional morphology of Machairodontine felids. Estudios Geológicos, 67, 255–276.

[ar25690-bib-0110] Ungar, P. S. (2010). Mammal teeth: Origin, evolution, and diversity. JHU Press.

[ar25690-bib-0111] Van Valkenburgh, B. (2007). Déjà vu: The evolution of feeding morphologies in the Carnivora. Integrative and Comparative Biology, 47, 147–163. 21672827 10.1093/icb/icm016

[ar25690-bib-0112] Van Valkenburgh, B. (2008). Costs of carnivory: Tooth fracture in Pleistocene and recent carnivorans. Biological Journal of the Linnean Society, 96, 68–81.

[ar25690-bib-0113] Van Valkenburgh, B. , & Hertel, F. (1993). Tough times at La Brea: Tooth breakage in large carnivores of the late Pleistocene. Science, 261(5120), 456–459. 10.1126/science.261.5120.456 17770024

[ar25690-bib-0114] Van Valkenburgh, B. , & Jenkins, I. (2002). Evolutionary patterns in the history of Permo‐Triassic and Cenozoic synapsid predators. The Paleontological Society Papers, 8, 267–288.

[ar25690-bib-0115] Van Valkenburgh, B. , & Ruff, C. (1987). Canine tooth strength and killing behaviour in large carnivores. Journal of Zoology, 212, 379–397.

[ar25690-bib-0116] Werdelin, L. , McDonald, H. G. , & Shaw, C. A. (2018). Smilodon: The iconic Sabertooth. JHU Press.

[ar25690-bib-0117] Wheeler, T. (2011). Experimental paleontology of the scimitar‐tooth and dirk‐tooth killing bites. In V. L. Naples , L. D. Martin , & J. P. Babiarz (Eds.), The other saber‐tooths: Scimitar‐tooth cats of the Western hemisphere. JHU Press.

[ar25690-bib-0118] Whitenack, L. B. , & Motta, P. J. (2010). Performance of shark teeth during puncture and draw: Implications for the mechanics of cutting. Biological Journal of the Linnean Society, 100, 271–286.

[ar25690-bib-0119] Whitney, M. , LeBlanc, A. , Reynolds, A. , & Brink, K. (2020). Convergent dental adaptations in the serrations of hypercarnivorous synapsids and dinosaurs. Biology Letters, 16, 20200750. 33321067 10.1098/rsbl.2020.0750 PMC7775981

[ar25690-bib-0120] Wroe, S. (2008). Cranial mechanics compared in extinct marsupial and extant African lions using a finite‐element approach. Journal of Zoology, 274, 332–339.

[ar25690-bib-0121] Wroe, S. , Chamoli, U. , Parr, W. C. , Clausen, P. , Ridgely, R. , & Witmer, L. (2013). Comparative biomechanical modeling of metatherian and placental saber‐tooths: A different kind of bite for an extreme pouched predator. PLoS One, 8, e66888. 23840547 10.1371/journal.pone.0066888 PMC3694156

[ar25690-bib-0122] Zhang, B. , & Anderson, P. S. (2024). How rate‐based stretchability of soft solids controls fracture morphology in dynamic conical puncture. International Journal of Impact Engineering, 187, 104911.

[ar25690-bib-0123] Zhang, B. , & Anderson, P. S. L. (2022). Modelling biological puncture: A mathematical framework for determining the energetics and scaling. Journal of the Royal Society Interface, 19, 20220559. 36259171 10.1098/rsif.2022.0559 PMC9579757

[ar25690-bib-0124] Zhang, B. , & Anderson, P. S. L. (2023). Investigation of the rate‐mediated form‐function relationship in biological puncture. Scientific Reports, 13, 12097. 37495672 10.1038/s41598-023-39092-8 PMC10372153

[ar25690-bib-0125] Zhang, B. , Baskota, B. , Chabain, J. J. , & Anderson, P. S. (2024). Curving expectations: The minimal impact of structural curvature in biological puncture mechanics. Science Advances, 10, eadp8157. 39141731 10.1126/sciadv.adp8157 PMC11323891

[ar25690-bib-0126] Zienkiewicz, O. C. , & Taylor, R. L. (2000). The finite element method: Vol. 1 the basics. Butterwirth‐Heinemann.

